# Engineering and validation of a novel lipid thin film for biomembrane modeling in lipophilicity determination of drugs and xenobiotics

**DOI:** 10.1186/1754-1611-3-14

**Published:** 2009-09-07

**Authors:** Sunday Olakunle Idowu, Morenikeji Ambali Adeyemo, Udochi Ihechiluru Ogbonna

**Affiliations:** 1 Department of Pharmaceutical Chemistry, Faculty of Pharmacy, University of Ibadan, Ibadan, Nigeria

## Abstract

**Background:**

Determination of lipophilicity as a tool for predicting pharmacokinetic molecular behavior is limited by the predictive power of available experimental models of the biomembrane. There is current interest, therefore, in models that accurately simulate the biomembrane structure and function. A novel bio-device; a lipid thin film, was engineered as an alternative approach to the previous use of hydrocarbon thin films in biomembrane modeling.

**Results:**

Retention behavior of four structurally diverse model compounds; 4-amino-3,5-dinitrobenzoic acid (ADBA), naproxen (NPX), nabumetone (NBT) and halofantrine (HF), representing 4 broad classes of varying molecular polarities and aqueous solubility behavior, was investigated on the lipid film, liquid paraffin, and octadecylsilane layers. Computational, thermodynamic and image analysis confirms the peculiar amphiphilic configuration of the lipid film. Effect of solute-type, layer-type and variables interactions on retention behavior was delineated by 2-way analysis of variance (ANOVA) and quantitative structure property relationships (QSPR). Validation of the lipid film was implemented by statistical correlation of a unique chromatographic metric with Log P (octanol/water) and several calculated molecular descriptors of bulk and solubility properties.

**Conclusion:**

The lipid film signifies a biomimetic artificial biological interface capable of both hydrophobic and specific electrostatic interactions. It captures the hydrophilic-lipophilic balance (HLB) in the determination of lipophilicity of molecules unlike the pure hydrocarbon film of the prior art. The potentials and performance of the bio-device gives the promise of its utility as a predictive analytic tool for early-stage drug discovery science.

## Background

It is now well recognized that the molecular organization of biological membranes and their interactions with the extracellular and intracellular spaces are critical determinants of cell function. These features are essential to molecular medicine, critical to understanding of toxin mechanisms and mechanisms of drug action. However, the intrinsic complexity of the cell membrane system, a very specific molecular architecture of phospholipids, sterols, glycolipids, and proteins often precludes direct access to these features. This fact drives the development of simpler model systems that are more amenable to detailed characterization [1]

Lipophilicity is one of the most important physicochemical properties that affect the safety and efficacy of drug molecules and its measurement is commonly used to model the pharmacokinetic properties of drugs in drug discovery research [2]. The most commonly used parameter of lipophilicity, logarithm of octanol-water partition coefficient (Log P) is well documented in literature [3].

This conventional approach has been criticized as simplistic and tedious. Variety of chromatographic techniques has therefore been devised as alternative models. Reversed phase thin layer chromatography (RPTLC) and reversed-phase high performance liquid chromatography (RP-HPLC) are popular examples. Owing to the versatility of chromatographic methods and their amenability to high-throughput screening of prospective drugs, they have largely supplanted the traditional 'shake flask' method based on octanolwater partitioning. The main weakness of the various chromatographic models however, is that the stationary phase is invariably pure hydrocarbon in nature (e.g. liquid paraffin, octylsilane (C8), or octadecylsilane (C18) bonded phase), contrary to the amphiphilic chemistry of biological membrane [4-10].

The accuracy of prediction, based on *in vitro *data, of absorption, distribution, metabolism, excretion and toxicity (ADMET) characteristics is limited by this fact. The inaccuracies of predicted pharmacokinetic behavior often lead to failure of drug candidates in the early phases of clinical trials. Phospholipids are the most abundant lipid in the biological membrane; additional types of lipid often found are cholesterols, and glycolipid. Further biomembrane structural complexity is contributed by integral and peripheral proteins, bound to the membrane in varying degrees [11]. Simulation of the biomembrane by incorporating phsophatidycholine tags on silica support was therefore hypothesized to be a more realistic stationary phase for membrane permeability modeling *in vitro*. Immobilized artificial membrane (IAM) chromatography columns which implemented this design in liquid chromatography (LC) have been shown to correlate drug permeability data [12-16].

Kaliszan et.al [2], reviewed the applications of new stationary phases in lipophilicity determination. The newer phases include; monolithic silica columns, monoliths based on polystyrenes, polymethacrylates and polyacrylamides. Other examples are alkylamides, and those with different polar and nonpolar functional groups. Some other variants of chromatographic methods employ micellar phase. These methods include micellar electrokinetic chromatography (MEKC), microemulsion electrokinetic chromatography (MEEKC), and micellar liquid chromatography (MLC). Micelles and the newer silica-based stationary phases are amphiphilic in nature, and are therefore structurally closer to biomembrane than to octanol or the regular RP-HPLC stationary phase materials. A review of experimental and computational models of predicting absorption from physicochemical parameters, with emphasis on their relative merits has also being published [17].

Despite the attempts to mimic the biomembrane function by simulating amphiphilic chemistry of the most abundant lipid; phospholipids, the fact remains that the intrinsic complexity of the cell membrane system makes the modeling of the biomembrane practically challenging. A recent study reported a comprehensive molecular-level structural and functional characterization of the biomembrane architecture. The optimized design investigated is a sparsely tethered bilayer lipid membrane system that is chemically tethered to the solid support by a synthetic lipid [1]. The membrane systems are reportedly highly flexible and robust biomimetic system that is potentially useful for studies and applications of biological membranes.

To the best of our knowledge, there has been no report on the use of IAM-type or any amphiphilic membrane system in planar chromatography. The latter has the attractive feature of simplicity and versatility. We hypothesize that a naturally occurring lipid which is mainly triacylglycerol in nature would provide hydrocarbon moieties and acyl esters as polar heads and thus are closer to the diacyl esters and hydrocarbon moieties in phospholipids. In addition, the presence of lecithin in the lipid will afford incorporation of phosphate esters into any solid-supported layer prepared as stationary phase in RPTLC mode. A layer obtained from such a lipid should thus be a simulation of the biomembrane architectural complexity than the hydrocarbons previously used in planar chromatography. Literature search reveals that the fixed oil obtained from the seed of *Leucaena leucocephala *(Leguminosae, Lam. de Wit) is reportedly the richest vegetable source of phosphatides (lecithin) [18]. In addition, the oil was reported to contain sterols [19], tocopherols [20], and glycolipids [21]. The oil therefore has the desirable complexity of composition required for testing our hypothesis.

The main lipid constituents of biological membranes are phospholipids (phosphoacyglcyerols) such as lecithin (phosphatidycholine), fats and steroids. The 2-position of lecithin is usually esterified by an unsaturated and the 1-position by a saturated fatty acid of the C16 (palmitic acid) or C18 (stearic acid) series. Double unsaturation in the 2-position is common (esterification by linolieic acid). Phosphatidyl ethanolamine and phosphatidylserine (both found in the cephalin of brain) are variations of the lecithin structure. Cholesterol is the most abundant steroid [22,23]. Lipid compositions of leucaena oil show some similarities to the composition of lipid membrane. Detailed characterization of leucaena oil reveals the presence of approximately 26-29% saturated acids and 71-73% of unsaturated acids. The oil is rich in linoleic acid (42.5-65%), arachidic (0.8-1.6%) and lignoceric acid (0.7-1.7%). The main sterol is *β*-sitosterol(55%) [19,24,25]

This paper reports the refinement and optimization of the crude leucaena oil to obtain technical grade oil for routine use in engineering a planar thin film for biomembrane modeling. The crude and refined oil were also characterized by standard physicochemical properties, in order to delineate the impact of purification process on oil composition. Optimal lipid film thickness was designed by using liquid paraffin (LP) film on silica support as benchmark. The surface chemistry of the lipid film based on refined leucaena oil (LO) was evaluated by computational analysis, thermodynamic analysis and digital image analysis of the lipid layer under ultraviolet light (254 and 365 nm). Validation of the lipid thin film was implemented by statistical correlation of a derived chromatographic metric, which uniquely ranked the lipophilicity of the model compounds, with Log P (octanol/water) and several calculated molecular descriptors.

## Methods

### Equipment

Analytical balance (Mettler, H80, U.K.), Abbe refractometer (Abbe, 300778, England), vacuum pump (Edward, England), vacuum oven (Gallemkamp, England), Ultraviolet lamp (254 and 365 nm, Gallenkamp, U.K.),

### Chemicals

Potassium hydroxide, mercury II iodide, hydrochloric acid, phenolphthalein, sodium thiosulphate, sodium carbonate, wij's solution (iodine monochloride solution), chloroform, anhydrous pyridine, activated charcoal, silica gel (sorbent, thin layer chromatography grade, GF_254_), n-hexane, diethylether, acetone, carbon tetrachloride (all are British Drug Houses (BDH), U.K., analytical grade), liquid paraffin (LP, Moko, Lagos, Nigeria), thin layer chromatography aluminum sheets, 5 × 10 cm (0.2 mm, silica gel 60, F_254_, Merck, Darmstadt, Germany), nabumetone (Sigma, USA), naproxen (isolated and recrystallized from tablets), halofantrine (Glaxo Smith Kline, Lagos), 4-amino-3,5-dinitrobenzoic acid (ADBA, synthesized in our laboratory [26].

### Plant Material

The dried seed pods were collected from the plant *Leucaena leucocephala *(Lam.) de Wit. growing around Obafemi Awolowo Hall area, University of Ibadan and authenticated at Botany Department, University of Ibadan, Ibadan, Nigeria. The seeds were sun dried and pulverized with a milling machine.

### Oil extraction

The powdered seeds were weighed (3.2 kg) and packed in a cotton cloth bag and defatted by cold maceration in n-hexane. The marc was extracted continuously until the marc was exhausted. The crude oil was recovered by distillation and the combined oil residue was dried *in vacuo *at 40°C in a vacuum oven for 24 hours. About 80% of the total volume obtained was decanted, filtered through glass wool and stored for future use, while the yellowish sediments were discarded.

### Enhancement of crude oil

#### i Preparation of silica gel cartridges

Silica gel solid phase extraction (SPE) cartridges were prepared in the laboratory by packing silica gel (thin layer chromatography grade, 3 g), with the aid of vacuum line, into a 10 ml plastic injection syringe barrel, with a cotton wool plug.

#### ii Preparation of activated charcoal cartridges

Activated charcoal SPE's were prepared in the laboratory by packing activated charcoal (2 g), with the aid of vacuum line, into a 10 ml plastic injection syringe barrel with cotton wool plug.

#### iii Optimization of the purification protocol

Serial dilutions of the crude oil in *n*-hexane; 50, 25, 20, 10, and 5% (v/v) were prepared as stock solutions. Each of the stock solutions (25 mL) was filtered, in turn, through 1 silica gel and 1 activated charcoal cartridges arranged in tandem. The hexane in the filtrate was distilled off to recover the oil. The recovered oil was centrifuged at 2500 rpm for 15 minutes and decanted. The final product was dried at 40°C in vacuum oven for 24 hours.

#### iv Endpoint of oil refinement

Thin layer chromatography plate (5 × 10 aluminum sheets, 0.2 mm, silica gel 60 F_254_, E. Merck, Darmstadt, Germany) was coated by ascending development in a solution (5% v/v) of the oil (crude sample and every batch of purified sample) in *n*-hexane. The plates were inspected after development for the presence of a yellow band of pigment at the solvent front. The highest concentration of the crude oil stock solution that produce a refined oil sample which gave a lipid-coated plate with no visible band at the solvent front was selected as the optimal stock solution for the routine purification of the oil.

### Determination of physicochemical properties

Some physicochemical properties of the oil were determined for the crude oil and the refined oil obtained by filtering 25 mL of the crude oil stock solution (10% v/v) through the assembly of silica gel and charcoal SPE cartridges as described above. The physicochemical properties determined as previously described [27] are saponification value, iodine value, peroxide value, acid value, ester value, hydroxyl value, relative density, and refractive index.

### Optimization of lipid film thickness

#### i. Preparation of LP benchmark and LO alternative layers

Liquid paraffin in *n*-hexane (5% w/v) was used as coating solution of silica gel support, to produce the layer which was taken as benchmark. Thin film of liquid paraffin was placed on the silica surface by ascending development of the TLC plates in the coating solution and allowing the plates to air dry afterwards. Alternative silica-supported LO lipid films were prepared by varying the concentration of LO coating solution (1.25, 2.50, 3.75, and 5.0% v/v).

#### ii Engineering of LO optimal film thickness

The optimal film thickness was designed by testing the alternative films against array of LP benchmark requirements and constraint. Using nabumetone as model compound and varying the concentration of organic modifier (methanol) in the mobile phase, a plot of linear regression of *Rm *against methanol fraction was made for LP and all alternative LO layers. *Rm *was obtained from the expression  where *R*_*f *_is the chromatographic retardation factor [5]. Retention behavior of nabumetone on LP was compared with its behavior on the alternative LO layers by statistical comparison of the slope (*S*, specific hydrophobic surface area) and y-intercept, (*Rm*_*w*_, basic lipophilicity parameter) of the regression plot of Rm versus methanol fraction. The retention behavior is described by the regression equation: *Rm *= *Sφ *+ *Rm*_*w *_(where *φ *is the methanol fraction). Plate development cycle time for a specific methanol fraction (0.5) was set as design constraint. This was compared by recording the time required for the mobile phase (50% methanol) to traverse 7 cm path length of the various silica-supported layers.

### Evaluation of surface chemistry- digital image analysis

The silica-supported lipid layer obtained by coating the TLC plate in a solution of the refined leucaena oil (LO) in hexane (3.75% v/v) was visualized under ultraviolet lamp at 254 and 365 nm. Digital images of the lipid layer under the UV light were recorded by (fluorescent-H mode, flash off, ISO 400) of a digital camera (IXY Digital 500, Canon Inc., Japan) and edited with a photo editing software Photoimpression 5 (Arcsoft, Inc., USA). Image analysis was carried out to provide insights into molecular differentiation of the lipid surface and possible relationship with the structure and function of biological membrane.

### Validation of engineered lipid thin film for membrane permeability modeling

The engineered lipid film was validated by comparing its performance with the prior art; liquid paraffin film [4,5] and octadecysilane (ODS) bonded phase [12-15], which was selected as reference. Using 4 model compounds; 4-amino-3,5-dinitrobenzoic acid (ADBA), naproxen (NPX), nabumetone (NBT) and halofantrine (HF), the impact of simulating the biomembrane phospholipids chemistry in the *in vitro *technique employed for determining lipophilicity descriptors for drug molecules was sought.

The computational model employed is a transformation of the planar chromatographic retardation factor *R*_*f *_according to the following equation:

(1)

Linear regression of the *Rm *values against the fraction of methanol organic modifier in the mobile phase was performed according to the equation:

(2)

S, is the specific hydrophobic surface area, while *Rm*_*w *_is the basic lipophilicity parameter calculated from the chromatographic data. The ultimate derived lipophilicity parameter of interest is the isocratic chromatographic hydrophobicity index (*φ*_*o*_), which signifies the methanol fraction that will produce half-maximum solute migration on the chromatogram for each compound. It is derived from the regression equation as *φ*_*o *_= -*Rm*_*w*_/*S *(where *φ*_*o *_is the value for *φ *when Rm = 0). Derived lipophilicity parameter (*φ*_*o*_) values were correlated with Log P and several ChemSketch calculated molecular descriptors to provide quantitative structure property relationships (QSPR) as previously described [5].

### Statistical analysis

The relative merit of various LO film thickness in comparison with LP benchmark was determined by 1-way analysis of variance (ANOVA) and Dunnet's multiple comparison tests. Significant difference was taken as *p < 0.05*. These tests were performed on both the slope (S) and the y-intercept (*Rm*_*w*_). The retention behavior of the 4 model compounds on 3 layer types was investigated by linear regression of *Rm *against methanol fraction. The effect of the two variables; solute-type and hydrophobic layer-type as well as possible interaction of the two on chromatographic determination of lipophilicity was evaluated by 2-way analysis of variance (ANOVA), using the two determined hydrophobic parameters (*Rm*_*w *_and S) as quality and performance attributes. QSPR was sought by correlation of *φ*_*o *_against calculated molecular descriptors as well as Log P for model compounds. The goodness of fit was measured by the coefficient of determination (*R*^2^) and standard deviation of y residuals (*S*_*y*,*x*_) (Graphpad Prism, SanDiego, CA)

## Results

The crude oil is dark brown with greenish yellow tint (5% yield). The optimal purification protocol is the filtration of 25 mL of crude oil in n-hexane (10% v/v) through 1 each of the silica gel and charcoal SPE cartridges in succession. The cartridges are effective for sample clean up, retaining much of the yellow pigments and the dissolved secondary metabolites. The refined oil is pale yellow in color, freed of colloidal particles by centrifugation. Drying in vacuum oven effectively removes last traces of *n*-hexane, while absence of yellow band on the solvent front of the chromatoplate signifies substantial removal of pigments and very fine particulate components of the oil (77% yield). Physicochemical properties determined for the crude and refined oil are shown in Table 1. The iodine value of both crude and refined oil is low and similar. Refined oil has lower hydroxyl value but higher acid and ester values.

**Table 1:** Physicochemical properties of crude and refined seed oil of *L. leucocephala*

**Parameters**	**Crude oil**	**Refined oil**
Saponification value	203.15	233.52
Iodine value	12.53	11.99
Peroxide value	0	0
Acid value	5.61	11.55
Ester value	197.54	221.97
Hydroxyl value	98.46	42.41
Relative density	0.9241	0.9254
Refractive index	1.4700	1.4700

**Figure 1:**
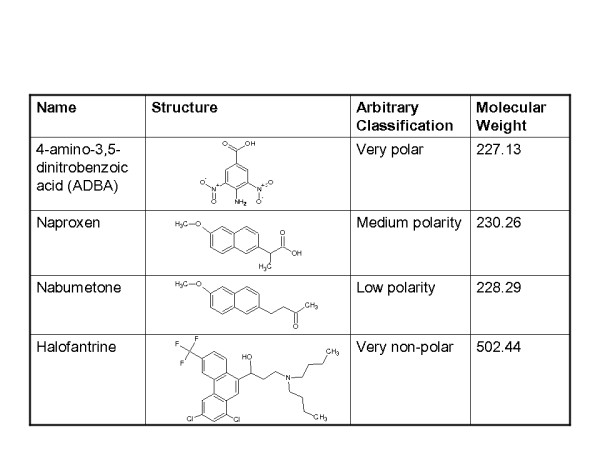
**Chemical structures of model compounds**.

The chemical structure of nabumetone is shown in Figure 1. Chromatographic hydrophobic parameters deduced from the linear regression of *Rm *for nabumetone versus methanol fraction on LP and alternative LO layers are shown in Table 2.

**Table 2:** Hydrophobicity parameters *Rm*_*w*_, *S *and *φ*_*o *_obtained for nabumetone on lipid films of various thicknesses, highlighting benchmark requirements and constraint

Layer type*	***S***	***Rm*_*w*_**	***φ*_o_**	***φ***	***n***	***R*^2^**	***S*_*y*,*x*_**
5% LP	-3.48	2.43	0.70	0.35-0.70	5	0.986	0.056
5% LO	-4.67	3.48	0.75	0.60-0.80	5	0.949	0.085
3.75% LO ^*a*^	-3.95	2.58	0.66	0.55-0.70	5	0.895	0.082
2.5% LO	-3.14	1.94	0.62	0.50-0.70	5	0.974	0.040
1.25% LO ^*b*^	-2.33	1.05	0.45	0.25-0.60	5	0.981	0.047

*LP = liquid paraffin, LO = leucaena oil,

^a^Statistically similar to benchmark (LP, p > 0.05, 1-way ANOVA)

^b^Plate development cycle time (for 7 cm path length and *φ *of 0.5) is 39 min, lower than the range 45-50 min for other film thicknesses investigated.

**Figure 2:**
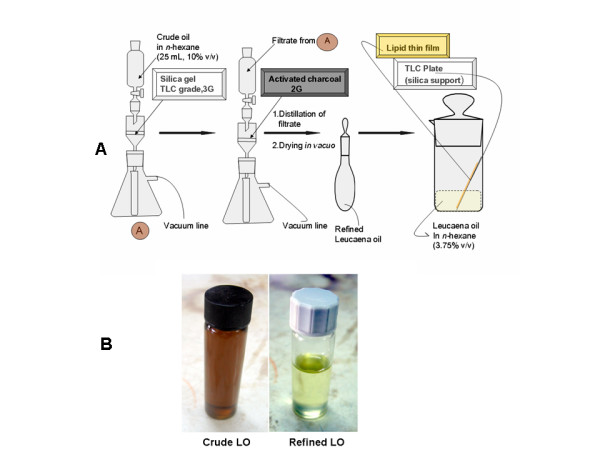
**(A) Schematic representation of the purification of leucaena oil and creation of the lipid thin film, (B) samples of crude and refined leucaena oil**.

**Figure 3:**
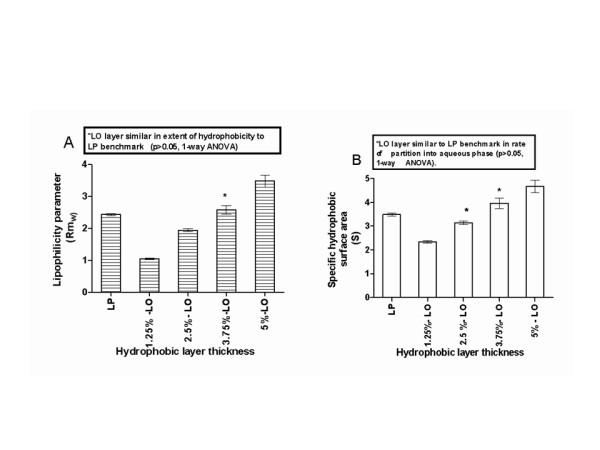
**Optimization of leucaena oil (LO) film thickness relative to liquid paraffin benchmark film, using (A) surface hydrophobicity and (B) rate of partition into aqueous phase as benchmark requirements**.

**Figure 4:**
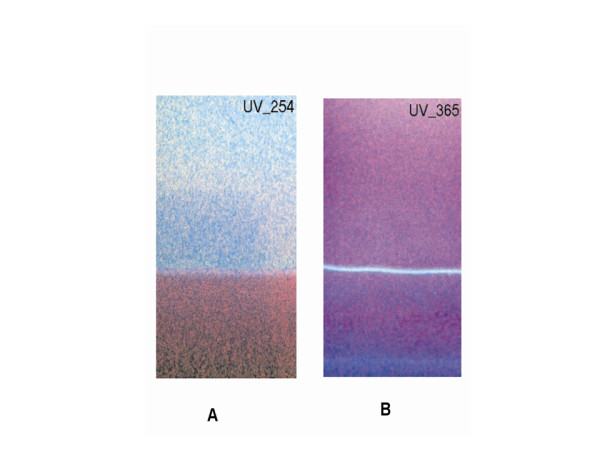
**Surface images of the silica--supported lucaena oil (LO) lipid film under ultraviolet light at (A) 254 nm and (B) 365 nm showing two distinct zones**.

**Figure 5:**
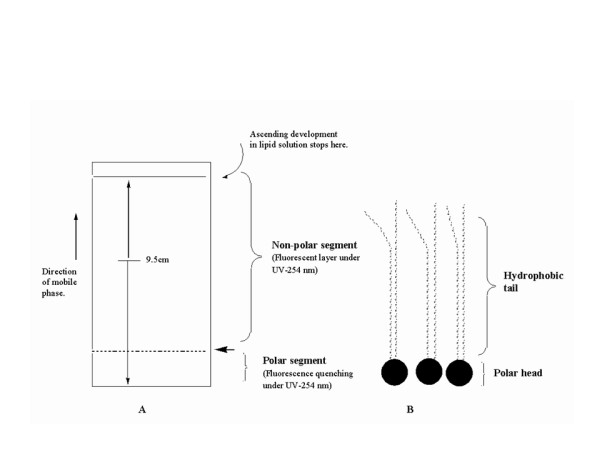
**Schematic representation showing molecular differentiation of (A) leucaena oil (LO) lipid film as a mimic of (B) amphiphilic structural configuration of phospholipid monolayer of the biological membrane, and hence a biomimietic artificial biological interface (ABI) model**.

**Figure 6:**
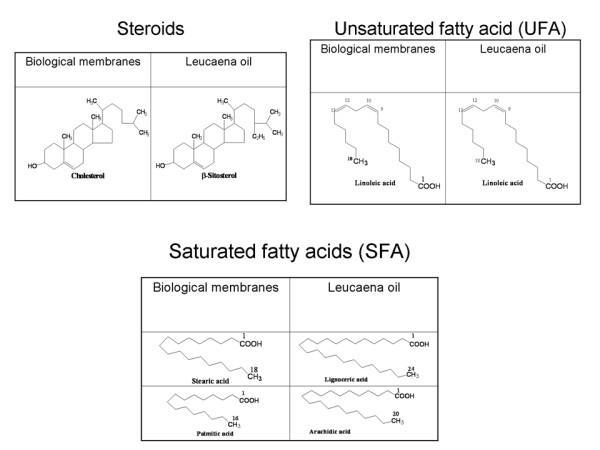
**Chemical structures of lipids found in biological membranes and leucaena oil showing similarities of lipid composition**.

A schematic representation of the process of refining leucaena oil and creation of lipid thin film is shown in Figure 2. The graphic representation of the multiple comparison of slope and y-intercept of the regression lines and the statistical inferences are shown in Figure 3. The digital images of the lipid film under UV-254 and 365 are shown in Figure 4. Schematic representation of molecular differentiation of the lipid layer, as revealed by image analysis and postulated relationship with the biomembrane structure is shown in Figure 5. Figure 6 shows the chemical structures of specific fatty acids and steroids found in leucaena oil and most biological membranes, highlighting the similarities in lipid compositions.

**Figure 7:**
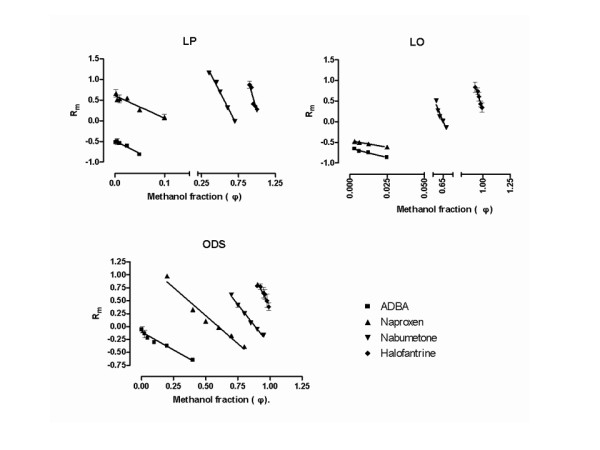
**Linear relationships between Rm and methanol fraction showing retention behavior of model compounds on liquid paraffin film (LP), leucaena oil film (LP) and octadecylsilane (ODS) layer**.

**Figure 8:**
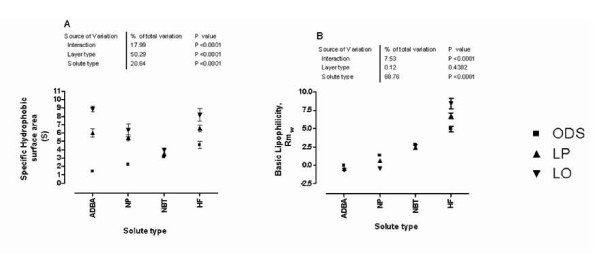
**Retention behavior of: (A) ADBA exemplifies stronger solute-layer hydrophobic interaction on pure hydrocarbon layers (LP and ODS) relative to a layer incorporating polar heads (LO), (B) HF exemplifies the interaction between the two variables; layer type and solute type in the determination of lipophilicity**.

**Figure 9:**
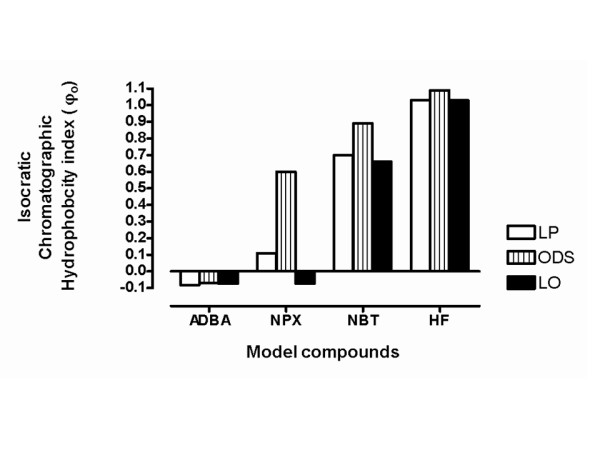
**Anomalous retention behavior shown by the value and sign of *φ*_*o *_for naproxen as determined by lipid layer (LO) relative to the estimate on pure hydrocarbon layers (LP and ODS) underscores the difference in partition mechanism at the two categories of interface**.

The linear regression of Rm against methanol fraction showing the retention behavior of the 4 model compounds on the 3 layers, namely; LP film, LO film and ODS layer is shown in Figure 7, while the hydrophobic parameters deduced from these plots are shown in Table 3. The overall impact of solute type (relative polarity) and layer type (hydrocarbon or biomimetic) and interaction between the two variables, on the hydrophobic parameters are displayed in Figure 8. Partition dynamics on LO film and the pure hydrocarbon layers (LP and ODS) follow different mechanisms, as revealed by the estimation of lipophilicity of naproxen on the 3 layers (Figure 9). The results of the calculated molecular descriptors and the correlation found with Log P (octanol/water) and *φ*_*o *_determined on the various layer types are shown in Table 4.

**Table 3:** Basic and derived lipophilicity parameters for model compounds on 3 layer types.

**Layer type**	***S***	***Rm*_*w*_**	***φ*_*o*_**	***φ***	***n***	***R*^2^**	***S*_*y*,*x*_**
ADBA							

LO	-8.84	-0.646	-0.073	0.003125-0.025	4	0.974	0.0141
LP	-5.99	-4.85	-0.081	0.0025-0.0254	4	0.806	0.0495
ODS	-1.41	-0.102	-0.073	0.025-0.40	5	0.915	0.0620

Naproxen							

LO	-6.32	-0.459	-0.073	0.003125-0.025	4	0.753	0.0350
LP	-5.42	0.610	0.11	0.0025-0.10	6	0.819	0.0959
ODS	-2.18	1.30	0.60	0.20-0.80	6	0.961	0.0944

Nabumetone							

LO	-3.95	2.58	0.66	0.55-0.70	5	0.895	0.0815
LP	-3.48	2.43	0.70	0.35-0.70	5	0.986	0.0556
ODS	-3.13	2.77	0.89	0.70-0.95	6	0.978	0.0438

Halofantrine							

LO	-8.13	8.40	1.03	0.925-0.99	5	0.738	0.120
LP	-6.54	6.76	1.03	0.90-1.0	5	0.864	0.102
ODS	-4.55	4.94	1.09	0.90-0.975	6	0.665	0.107

**Table 4:** Calculated molecular descriptors and goodness-of-correlation of derived hydrophobicity descriptor (*φ*_*o*_) with calculated molecular descriptors.

**S/No**	**Theoretical molecular descriptors/Model compounds**					**Coefficient of determination (*R*^2^) in correlation of *φ*_*o *_with calculated descriptors**		
	
		**ADBA**	**NPX**	**NBT**	**HF**	**LP**	**ODS**	**LO**
1.	Refractive index	1.719	1.608	1.575	1.562	0.720	0.980	0.577
2.	Surface tension(dyne/cm)	101.6	47.4	39.8	44.6	0.505	0.869	0.360
3.	Density (g/cm^3^)	1.775	1.197	1.084	1.25	0.398	0.766	0.267
4.	Polarizability (10^-24 ^cm^3^)	20.02	26.37	27.59	51.42	0.747	0.608	0.723
5.	Parachor (cm^3^)	406.2	504.6	529	1033.1	0.721	0.557	0.709
6.	Molar refractivity (cm^3^)	50.51	66.52	69.61	129.7	0.747	0.608	0.723
7.	Molar volume (cm^3^)	127.9	192.2	210.5	399.7	0.791	0.672	0.757

*Experimental data*								

1.	Log P*_(octanol/water)_	-1.50	3.18	3.27	3.25	0.468	0.852	0.32
2. a	*φ*_*o *_LP	-0.081	0.11	0.70	1.03	-	-	-
b.	*φ*_*o *_LO	-0.073	-0.073	0.66	1.03	-	-	-
c.	*φ*_*o *_*ODS*	-0.073	0.6	0.89	1.09	-	-	-

* Log P values were taken from literature

## Discussion

The purified oil has an iodine value of 11.99, which is comparable to 12.53 of the crude oil. This is more than 10 times lower than 132, the reported iodine value of soya bean oil. In addition, the peroxide value of freshly prepared sample is 0, as opposed to 0.5 for Soya bean oil [28]. This suggests the oil is relatively stable. The relative stability of the oil is an attractive feature for the intended technical purpose of engineering a lipid film with a large surface area. Presence of residual amounts of antioxidant substances like polyphenols known to be found in the seed could contribute to lipid stability. The zero peroxide value obtained for the oil underscores the stability and corroborates the interpretation of iodine values.

Higher acid and ester values obtained for the refined oil suggests purification enriched the oil with carboxylic acids and esters possibly by the removal of certain basic counter ions (e.g. alkaloids) and other secondary metabolites in general. Removal of flavonoids and other polyphenols could lead to the decrease in hydroxyl value found for the purified oil. This assertion is corroborated by the fact that we have previously reported the large presence of alkaloids, and polyphenols in the ethanol extract of the seed [29]. Other physicochemical properties are quite similar between the crude and purified oil.

The choice of 5% solution of the refined oil in hexane for preparing the lipid layer used for monitoring purification end point was suggested by prior art. Liquid paraffin layer is widely employed for membrane permeability modeling by planar chromatography, and the layer is usually prepared by plate development in 5% solution of liquid paraffin in hexane [4,5]. Although the lipid layer thickness and extent of hydrophobicity obtained is not necessarily equivalent to that produced by liquid paraffin, using this concentration helps to optimize the composition of the refined oil such that it is devoid of particulate matters that can be filtered on the silica gel plate surface.

### Design of an optimal silica-supported LO film

#### Computational analysis

The choice of nabumetone as the model compound for optimizing the surface hydrophobicity of the LO lipid film and rate of partitioning into the aqueous phase was based on the presence of an aromatic hydrophobic core of naphthalene residue. This was to ensure hydrophobic interaction as the dominant factor. A further consideration was the absence of any hydrophilic substituent that is capable of hydrogen bond donation. The ether linkage and the carbonyl function in the side chains are both capable of hydrogen bond acceptance alone. The impact of specific interactions is therefore minimized while hydrophobic interaction was maximized in the partition dynamics employed for optimizing the LO film thickness.

Coating the plates in a solution of LO in hexane (3.75% v/v) was concluded on as the recommended procedure for the design of optimal film thickness for subsequent routine use. This inference was made because Dunnet's test shows that both the slope (S, measure of rate of partition into the aqueous phase) and y-intercept (extent of hydrophobicity of the lipid film) obtained on this particular layer, were statistically similar (p > 0.05) to the LP benchmark values. The thin film obtained by coating the plate in 2.5% LO in hexane, gave a slope that is statistically similar to the LP layer but the y-intercept was statistically different (p < 0.05, Figure 3). This statistical difference is of practical significance, because the intercept signifies surface hydrophobicity of the layer. The latter is a critical parameter for the proposed artificial bio-interface function. Film thickness obtained by coating the plate in 1.25 and 5% LO in hexane are significantly different in both the slope and y-intercept (p < 0.01) relative to benchmark regression parameter values. The optimal film thickness was thus designed with some statistical rigor and reliance on a previously validated benchmark.

#### Image analysis

The surface image of the lipid film under UV-254 nm shows two distinct zones (Figure 4a). The dark lower zone is due to fluorescence quenching on account of the presence of UV active constituents in the triglyceride lipid matrix. This segment is composed of more polar constituents of the lipid and hence more highly retained by the polar silica gel support. The chromophoric lower zone is therefore a self-assembly of relatively polar molecules in their descending order of polarity, from the base of the plate to the boundary of the two zones.

The surface image of the lipid film under UV-365 nm has a different appearance and does not show as much contrast of the 2 zones, because the pre-coated silica gel plate has a fluorescent additive that fluoresces when exposed to 254 nm wavelength in particular (i.e. silica gel 60 F_254_). The narrow fluorescent band observed on the layer (Figure 4b) therefore signifies the spectroscopic behavior of a particular class of compound in the lower region of the lipid layer. Further expositions of molecular differentiation of the lower zone and chemical fingerprinting by spectroscopic means is warranted, in order to obtain mechanistic comprehension of the partition process at the engineered bio-interface.

In this proposed *in vitro *model, the analytes will be spotted on the polar segment of the layer which will be in contact with the aqueous phase. During chromatographic development, solutes will traverse the polar segment before moving to the more hydrophobic segment composed of triglycerides, which are made up of 3 acyl ester functionality per mole of glycerol. This chemistry is closer to the 2 acyl ester functionality per mole of glycerol found in the hydrophobic moiety of biomembrane phospholipids and a significant departure from the pure hydrocarbon layers (e.g. liquid paraffin and ODS) previously employed in reversed-phase planar chromatography methods. The LO planar lipid film is therefore, potentially, a more realistic model of biomembrane structure and function. It represents a biomimetic artificial biological interface (ABI), and may be useful for membrane permeability modeling.

### Validation of artificial biological interface model

#### Specific hydrophobic surface area (S)

S is the specific hydrophobic surface area. It is otherwise descriptive of the rate of partition of the solute into the aqueous phase from the stationary lipoid phase. Pattern-recognition analysis shows that layer type accounts for the largest percentage of total variation in the values of S (>50%, p < 0.0001). This underscores the fact that layer-type significantly influence the mechanism of partition. Across the board, LO layer gave the highest S value. This implies LO layer retains the solutes by specific interaction, in addition to hydrophobic interaction. The involvement of specific interaction facilitates partition into aqueous medium by similar specific interaction, i.e. dipole-dipole (hydrogen bonding) between water molecules and the solutes on one hand, and solute-layer interaction on the other. The variation also shows significant interaction between the layer and solute types, such that interaction accounts for 18% of total variation (p < 0.0001). The impact of layer type on S varies with the capability of the solute for hydrogen bond formation. NBT that has capacity for hydrogen bond acceptance but not donation gives a cluster of S value on the 3 layers. On the other hand, ADBA with extensive capacity for hydrogen bond donation and acceptance shows the widest scatter (Figure 8a). Solute type therefore contributes significantly to the overall variation, accounting for 20.64% of the total variation (p < 0.0001).

#### Basic lipophilicity paratmeter (Rmw)

The solute type is a significant factor accounting for 89% of the total variation in *Rm*_*w *_values (p < 0.0001). This shows the basic lipophilicity of the 4 model compounds are different, confirming the arbitrary classification of the solutes into four broad classes - very polar, medium polarity, low polarity, very non-polar. The layer type however is not a significant factor in the variation of the *Rm*_*w *_values, this factor accounts for only 0.12% (p = 0.4382). This fact suggests that surface hydrophobicity of the 3 layers is equivalent, hence layer type does not affect the estimate of basic lipophilicity. This finding corroborates the prior optimization of LO layer thickness, which was designed with LP film as benchmark. Furthermore, the results confirm the similarity of surface hydrophobicity of the 2 layers prepared in the laboratory with that of the commercially available bonded phase ODS layer. Finally, statistically significant interaction also exists (7.53%, p < 0.0001) between the two variables; layer type and solute type. Retention behavior of HF exemplifies the interaction effect, because *Rm*_*w *_clusters around a value for the 3 other solutes, but shows a wider scatter for HF (Figure 8b)

#### Isocratic chromatographic hydrophobicity index (*φ*_*o*_)

The derived lipophilicity parameter *φ*_*o*_, was computed to estimate the lipophilicity of the solutes, and the *φ*_*o*_values are graphically displayed in Figure 9. *φ*_*o*_, which is a ratio of the two basic parameters, has been previously shown to be a more accurate estimate of lipophilicity than the basic lipopbilicity parameter, *Rm*_*w *_[5]. The pattern shows that LP and ODS gave a similar hydrophobicity ranking of the 4 model compounds. The sequence obtained is:



On the other hand, LO layer suggests a slightly different sequence:



The LO layer is here shown to rank the lipophilicity of naproxen to be closer to that of ADBA (in value and sign) and farther from nabumetone, unlike the ranking of LP and ODS.

Strong solute-layer hydrophobic interaction is revealed by a decrease in specific hydrophobic surface area (S). LO layer therefore exhibited the weakest hydrophobic interaction, since it shows the highest S value across the board. Given the demonstrated evidence that surface hydrophobicity of the 3 layers is similar, the significant difference shown in the value of S on the various layers for each solute type reflect the varying degree of hydrophobic interaction involvement in the partition mechanism. This pattern confirms the hypothesis that LO film, on account of polar heads - acyl and phosphate groups - should permit specific interactions. The extent of hydrophobic interaction exhibited by the layer follows the sequence:



S is therefore a solute-dependent measure of the interplay between solute-layer hydrophobic and specific solute-layer, solute-solvent interaction at the interface. Any molecule with *φ*_*o *_value > 1.0 should be adjudged very non-polar and would exhibit poor water solubility e.g. HF. On the contrary, any molecule with *φ*_*o *_value < 0 should be adjudged very polar and would exhibit free water solubility e.g. ADBA.

This interpretation of results is however limited to the use of methanol as organic modifier. Methanol has been shown to be the preferred organic modifier for chromatographic *Rm *studies, principally due to its closeness to water in chemistry. In the event a solute is so hydropbobic that even 100% methanol could not produce reasonable solute migration, other organic modifiers with larger hydrophobic moiety would be more appropriate e.g. acetone and dimethylformamide [5], in order to obtain sufficient experimental data.

Another important finding is that pure hydrocarbon layers like LP and ODS seem to overestimate the lipophilicity of molecules with hydrophilic fragments, once they have a large hydrophobic moiety. Naproxen is the relevant example of this scenario. The large naphthalene moiety dominates the estimation, while the effect of COOH in the side chain is minimized on ODS. Whereas, the presence of the highly hydrophilic COOH has a higher impact on LO partition dynamics. The latter can therefore be said to be more sensitive to the differences in polarity of the solutes and captures solute-layer specific interactions in the overall estimate of lipophilicity. The glaring difference in the estimate of lipophilicity of naproxen on LO relative to the two purely hydrocarbon layers (LP and ODS) is the unambiguous evidence (Figure 9). The practical significance of this feature in biomembrane models as exemplified by LO film is corroborated by the classical example shown by the lipophilic terfenadine and it's zwitterionic carboxylic acid derivative, fexofenadine. Terfenadine was withdrawn from clinical use because of cardiotoxicity, which is related to its high lipophilicity and biopartitioning profile. On the other hand, fexofenadine, which differ structurally by having a carboxyl group in place of a methyl group in terfenadine did not show the cardiotoxicity (QT prolongation) and also shows fewer over all side effects [30].

### Validation of model and QSPR

Theoretical molecular descriptors were calculated for the model compounds by ACD/ChemSketch version 8.0 (Advanced Chemistry Development, Inc. Ontario, Canada). Log P values were obtained from literature for ADBA [31]), NPX [32], NBT [33] and HF [34]. Quantitative structure property relationships (QSPR) were sought by correlation analysis. The goodness of fit of correlation between the calculated descriptors and the derived lipophilicity parameter *φ*_*o *_is shown by the coefficient of determination, *R*^2^, in Table 4.

The pattern reveals that *φ*_*o *_data generated on LO film gave better correlation with solubility properties, which are calculated basic macroscopic properties (e.g. molar refractivity and polarizability) than derived macroscopic properties (e.g. refractive index). The reverse of this pattern was obtained for ODS generated data, while LP did not give a clear distinction of pattern.

Log P values obtained from octanol/water partition shows low sensitivity to the hydrophilic-lipophilic balance (HLB) of molecules. This weakness is apparent in the cluster of Log P values reported for NPX, NBT and HF (Table 4). The values, albeit reported by different authors, even suggest NBT is more lipophilic than HF, which is a self-evident anomaly according to structural theory.

In the correlation of *φ*_*o *_with Log P values, the coefficient of determination follows the sequence: ODS > LP > LO, with *R*^2 ^values; 0.852, 0.468 and 0.321 respectively. This pattern suggests that the partition dynamics at the ODS-water and LP-water interface are closer to the partition dynamics in the rather simplistic octanol/water shake-flask method, than the LO water interface. Poor correlation of the LO data with Log P values corroborates the earlier deduction from our results that LO film is far more complex and the partition dynamics which is a simulation of *in vivo *biomembrane partition dynamics is a significant departure from the octanol/water system.

#### Thermodynamic analysis of biomembrane function modelling

For the interface partition process to be thermodynamically favorable, the Gibbs' energy of the partition process should be negative (Δ*G *< 0). The Gibbs energy is described by the relationship that follows; where SP is stationary phase and MP is mobile phase [5,35]:



For the overall expression to be negative, which signifies spontaneity of process, the expression *ΔG*_*o*(*gas→SP*) _should be positive. This in turn means Δ*H *(enthalpy) should be positive (endothermic) and Δ*S *(entropy change) should be negative, since



For Δ*S *to be <0, it means there must be more bond formation than bond breaking (bond formation decreases entropy) in the solute-layer interactions. Whereas for the Δ*H *to be >0 there must be more hydrogen bonds broken than are made. The requirements are therefore opposing.

Considering the 3 layer materials; ODS, LP, and LO, the latter is a biomimetic material and is shown to have acyl esters and phosphate esters, which are hydrogen bond acceptor sites in addition to the hydrogen bond donor site found in phosphatides and free fatty acids. The polar lower zone of LO film also comprises of chromophoric compounds with polar fragments that are capable of secondary bonding. LO film is therefore capable of hydrogen bond donation and acceptance, much as the aqueous mobile phase is capable of hydrogen bond formation and acceptance. Active sites on the LO film are therefore capable of retaining water molecules and thus discourage hydrophobic interaction.

This scenario readily leads to breaking of the clathrage cage of water molecules around the solute molecules spotted on the stationary phase, and Δ*H *is >0. The resulting increase in hydrophobic surface area, confirmed by experimental results, is accompanied by a large decrease in entropy owing to stronger dipole-dipole interaction of the solute with the solvent and the solvent molecules become more organized around the polar solutes, i.e. Δ*S*>0. These opposing requirements for thermodynamically favorable partition is readily achieved on LO film, because the large surface area is maintained. Presence of polar groups in the LO film surface discourages solute molecules from coming together in a single cavity as it is found in hdyrophobic interaction.

On the contrary, the purely hydrocarbon layers retain the solute mainly by hydrophobic interactions, which is accompanied by the formation of highly ordered clathrate cage of water molecules around the solutes. Partitioning into the aqueous phase will therefore necessarily require the breaking of bonds making up the clathrate cage to permit penetration of water molecules. For reasonable solute migration therefore, more hydrogen bond is broken than formed, i.e. Δ*H*>0. However, because the hydrophobic interaction is predominant on these layers, the solutes are readily pulled back into a single cavity, reducing the surface area, as epxerimental data confirms, thus releasing the ordered water molecules involved in solvation with a consequent increase in entropy (i.e. Δ*S*>0). The partition process is therefore less thermodynamically favorable.

In sum, the existence of specific electrostatic interactions alongside hydrophobic interactions on LO film interface results in a more thermodynamically favorable partition process than purely hydrocarbon layers. This scenario is a closer mimic of the function of the biomembrane, which involves interaction between hydrophobic and polar components of the lipid bilayer. It also underscores the validity of our postulated hypothesis that modeling biomembrane structure and function with a biomimetic material, is superior to the use of pure synthetic material (ODS) or mineral oil (LP). The mathematical treatment of partition dynamics of model compounds on three different interfaces as described here shows good agreement of deductions from the mathematical model with experimental data. This substantiates the fundamental power of mechanistic models in interpreting chemico-biological interactions. Recently, Dhurjati et.al., [36] pointed out the potential impact of systems biology, which is a synergistic interplay of mathematics and biology, on medicine. Systems level perspective was shown to be capable of novel and revolutionary impact on drug discovery. Our findings in this study corroborate this assertion.

## Conclusion

We have developed a simple and elegant artificial biological interface (ABI), specifically for modeling the biomembrane in lipophilicity determination of drugs and xenobiotics. The biodevice is potentially useful as a predictive analytic tool in early-stage drug discovery. Using liquid paraffin (LP) film as benchmark for RPTLC lipophilicity determination, an optimal silica-supported LO film was designed. The engineered lipid film incorporates hydrophobic moieties, free fatty acids, acyl and phosphate esters. This lipid film was validated by comparison of its performance with prior art like LP film and ODS bonded phase using a narrow range of structurally diverse model compounds representing 4 broad classeswith varying polarities.

Computational analysis utilized parameters that signify quality and performance attributes of the lipid film in a partition process for which the bio-device is intended. Surface image analysis provided supportive data to supplement computational analysis in characterizing the lipid film architecture. Thus, a combination of experimental and *in silico *methods was employed to design, optimize and validate the performance of the bio-device. Further validation studies are warranted to evaluate its performance over a wider range of drugs and xenobiotics. The relative merit of the lipid film and more traditional methods would also be assessed by comparing their correlation with membrane permeability data based on parallel artificial membrane permeability assay [37], which is the now popular alternative to caco-2 cell-line based assays.

Engineering of this novel device is biology-inspired, the ABI, like true biological interfaces, can therefore respond to stress, especially oxidative stress. The long term stability profiling of the lipid system on storage is therefore germane to reliability of results. These extensions of our findings are currently ongoing in the authors' laboratory.

## Abbreviations

ADBA: 4-amino-3,5-dinitrobenzoic acid; NPX: naproxen; NBT: nabumetone; HF: halofantrine; LO: leucaena oil; LP: liquid paraffin; ODS: octadecylsilane; TLC: thin layer chromatography; RP-TLC: reversed phase thin layer chromatography; RP-HPLC: reversed phase high performance liquid chromatography; ANOVA: analysis of variance; QSPR: quantitative structure property relationships; HLB: hydrophilic - lipophilic balance; ADMET: absorption, distribution, metabolism, excretion and toxicity; IAM: immobilized artificial membrane; MEKC: micellar electrokinetic chromatography; MLC: micellar liquid chromatography; MEEKC: microemulsion electrokinetic chromatography; SPE: solid phase extraction; ABI: artificial biological interface, *φ*, methanol fraction (used as mobile phase); *S*: specific hydropbobic surface area; *Rm*_*w*_: basic chromatographic lipophilicity parameter; *φ*_*o*_: isocratic chromatographic hydrophobicity index; *S*_*y*,*x*_: standard deviation of y-residuals; *R*^2^: coefficient of determination; *n*: number of data points in the linear regression.

## Competing interests

The authors declare that they have no competing interests.

## Authors' contributions

SOI made substantial contributions to original conception, experimental design, data analysis, data interpretation and manuscript writing. MAA and UIO were both involved in data acquisition.

## Authors' information

SOI was educated at Ibadan, Nigeria, and London (School of Pharmacy, Brunswick Square) and holds Bachelor of Pharmacy (B. Pharm.) degree and Ph.D. (Pharmaceutical Chemistry). He had post-doc training in Japan (Process Understanding and Modeling) and currently heads the Department of Pharmaceutical Chemistry, Faculty of Pharmacy, University of Ibadan, Nigeria. MAA holds B. Pharm. degree, while UIO holds B. Sc. (Chemistry) degree, they are both Research Students.
